# A Comprehensive Review of the Therapeutic Potential of Mucuna Pruriens

**DOI:** 10.3390/molecules31050868

**Published:** 2026-03-05

**Authors:** Zhan Bashev, Diana Karcheva-Bahchevanska, Raina Ardasheva, Stanislava Ivanova

**Affiliations:** 1 Department of Pharmacognosy and Pharmaceutical Chemistry, Faculty of Pharmacy, Medical University of Plovdiv, 4002 Plovdiv, Bulgaria; zhan.bashev@mu-plovdiv.bg (Z.B.); diana.karcheva@mu-plovdiv.bg (D.K.-B.); 2 Research Institute, Medical University of Plovdiv, 4002 Plovdiv, Bulgaria; 3 PERIMED-2, BG16RFPR002-1.014-0007, Central District, Vasil Aprilov Blvd. 15A, 4002 Plovdiv, Bulgaria; raina.ardasheva@mu-plovdiv.bg; 4 Department of Medical Physics and Biophysics, Faculty of Pharmacy, Medical University of Plovdiv, 4002 Plovdiv, Bulgaria

**Keywords:** *Mucuna pruriens*, velvet bean, phytochemistry, natural products, biological activity

## Abstract

*Mucuna pruriens* (L.) DC. (Fabaceae), commonly known as velvet bean, is an annual tropical legume widely distributed in India, Africa, and the Americas. It has a long history of use in traditional medicine for managing various health conditions. It is renowned for its anti-inflammatory, antimicrobial, aphrodisiac, and anti-Parkinson effects. The entire plant is considered health-promoting, particularly the seeds. They have been used for their neuroprotective, fertility-enhancing, and antioxidant effects. This review aims to compile all available information regarding the chemical composition of all parts of this medicinal plant. For this purpose, the complete databases of Google Scholar, Scopus, PubMed, and Web of Science available to date were utilized. All studies reported the presence of a diverse range of secondary metabolites, including phenolic compounds, such as phenolic acids, flavonoids, and tannins, as well as saponins and alkaloids. Most studies concentrated on the chemical characterization of the seeds, whereas the leaves, roots, and pods have received comparatively limited scientific attention. The seeds of *M. pruriens* are renowned for their high concentration of L-3,4-dihydroxyphenylalanine (L-DOPA), a metabolic precursor of dopamine. A large proportion of these studies originated from countries where *M. pruriens* naturally occurs. Few studies have been conducted on the chemical composition of velvet bean outside these regions. Despite the existing information on the chemical composition of *M. pruriens*. (seeds, leaves, and roots), further research beyond its natural habitat is required to gain a broader understanding of its chemical profile and pharmacological effects.

## 1. Introduction

**Figure 1:**
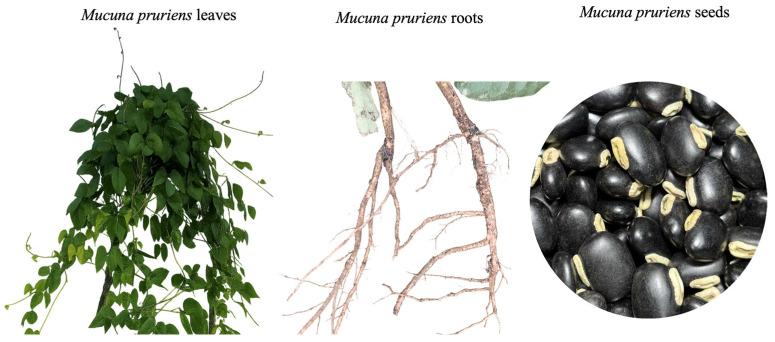
*M. pruriens* leaves, roots, and seeds.

*Mucuna pruriens* (L.) DC. (Figure 1) is a tropical legume belonging to the domain *Eukaryota*, kingdom *Plantae*, class *Magnoliopsida*, and order *Fabales*. It is classified within the family *Fabaceae* (Leguminosae), subfamily *Faboideae*, and tribe *Phaseoleae*. Species of *M. pruriens* are generally recognized to have three main botanical varieties. *M. pruriens var. pruriens* is the wild form of the plant. *M. pruriens* var. *utilis* is the most widely cultivated form [1]. *M. pruriens* var. *hirsuta* is also wild but has dense hairs on the pods. Commonly known as velvet bean, cowitch, cowhage, kivanch, kavach, or majram, *M. pruriens* is one of the most extensively studied species of the genus *Mucuna*, which comprises over 100 species. The species is diploid and widely distributed in tropical and subtropical regions. The plant is native to Africa, Asia, and America [2]. It is a twining, annual, self-pollinating, and herbaceous climber plant. The plant features long, slender stems with alternate, lance-shaped leaves and white blossoms that possess a bluish-purple, butterfly-like corolla. The pods are thick, leathery, and covered with dense, stiff hairs. Typically, they are approximately 10 cm in length and resemble violin sound holes, with each pod containing four to six dark brown seeds [2,3]. They can induce itching, a reaction attributed to the presence of histamine and 2-methylhistamine [4]. Due to its rich nutritional profile, particularly its high protein content, this species serves as an important dietary resource for both human consumption and livestock feeding [5]. Optimal growth occurs under warm and sunny conditions with adequate soil moisture. Like other members of the Fabaceae family, velvet bean engages in a symbiotic association with soil microbes that enables the fixation of atmospheric nitrogen. However, its cultivation has spread beyond its native habitat to other parts of the world [6].

Traditionally, *M. pruriens* has been valued in Ayurvedic and African medicine for its diverse pharmacological properties, including its ability to enhance fertility, mood, and neurological function. Because of their pleasant, coffee-like flavor, the seeds of this plant are used in Central America as a caffeine-free coffee substitute, where the drink is traditionally known as ‘Nescafé’ [7]. Primitive tribal groups on the northern coast of Andhra Pradesh, who call the plant “Duladundi,” have traditionally used *M. pruriens* in combination with other medicinal plants to treat tuberculosis [8].

Phytochemical analyses have revealed that *M. pruriens* contains a broad spectrum of bioactive compounds, such as alkaloids, flavonoids, saponins, tannins, and phenolic acids. However, its most notable constituent is L-DOPA, biogenetically formed via the shikimate pathway from the amino acid L-tyrosine through tyrosine hydroxylase, a direct precursor of dopamine, which has made the plant an important natural source for the management of Parkinson’s disease [3,9,10,11,12]. Dopamine is a crucial neurotransmitter that plays a fundamental role not only in the pathophysiology of Parkinson’s disease but also in a wide range of physiological and behavioral processes. These include memory, concentration, motivation, and hormonal regulation [13]. At low concentrations, dopamine may induce vasodilation, thereby enhancing the blood circulation. Furthermore, it is involved in the regulation of obesity, sexual behavior, and mood [14,15,16].

In recent decades, research has intensified at both experimental and clinical levels to validate traditional claims and explore new pharmacological applications of *M. pruriens*. Animal and human studies have demonstrated its potential in improving male reproductive health, reducing oxidative stress, modulating endocrine functions, and alleviating neurodegenerative symptoms.

Although the plant exhibits numerous beneficial effects on the human body, its consumption should be approached with caution. Thermal processing or fermentation of the seeds effectively inactivates most anti-nutritional factors. However, excessive levels of secondary metabolites, particularly L-DOPA, may pose a risk of toxicity to the consumer.

The present review aims to summarize current insights into the phytochemical composition, biological activities, and therapeutic potential of *M. pruriens*. Emphasis is placed on in vitro, in vivo, and human studies that elucidate the pharmacological actions and clinical relevance of this plant species. By consolidating the available data, this study seeks to provide a comprehensive understanding of *M. pruriens* as a promising candidate for future pharmacotherapeutic development.

## 2. Materials and Methods

A comprehensive literature search was performed across PubMed, Scopus, Web of Science, and Google Scholar. Search terms included “*Mucuna pruriens*”, “velvet bean”, “phytochemistry”, “L-DOPA”, and “biological activity”. Boolean operators (AND/OR) were applied to refine the results. No restrictions regarding publication year were implemented.

All identified records were screened in two phases: initial assessment of titles and abstracts and evaluation of full-text articles. Studies were included if they examined the phytochemical composition or biological activity of *M. pruriens*; reported in vitro, in vivo, or human clinical findings; provided sufficient methodological detail and analyzable data; and were published in peer-reviewed journals in English.

Exclusion criteria were non-peer-reviewed sources (blogs, popular media, and webinars); inadequately described methodologies; and a lack of quantitative or qualitative data.

Data from eligible studies were extracted manually and organized according to plant part studied, extraction method, and biological outcomes.

## 3. Results

A total of 108 studies met the inclusion criteria. Eleven studies were reviewed to gain a deeper understanding of the botanical characteristics and history of the use of the plant. Thirty publications examined the phytochemical composition of *M. pruriens*, predominantly focusing on the seeds, whereas considerably fewer investigations analyzed the leaves, roots, and pods. Essential oil composition was assessed in only two studies. The seeds were consistently reported to contain high levels of primary metabolites—including proteins, lipids, carbohydrates, vitamins, and amino acids—with particularly high protein content supporting their nutritional use in animal feed. Twenty-six in vitro studies and forty-one in vivo animal studies were identified, while human clinical evidence remained limited to nine publications. Most biological activity studies evaluated antioxidant, antimicrobial, antiparasitic, anti-inflammatory, cytotoxic, analgesic, aphrodisiac, and fertility-enhancing effects. Several clinical studies also reported improvements in Parkinson’s disease symptoms following preparations made from *M. pruriens*.

### 3.1. Chemical Composition

#### 3.1.1. Leaves

**Figure 2:**
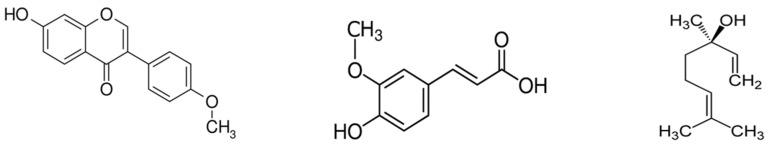
Chemical structures of formononetin, ferulic acid, and linalool detected in the leaves.

The phytochemical profiles summarized in Table 1 show that the leaves of *M. pruriens* consistently contain phenolic compounds, including anthraquinones, flavonoids, and tannins, in addition to alkaloids, saponins, and carbohydrates. The chemical structures of several of the most frequently identified compounds in the leaves are illustrated in Figure 2. The essential oil (EO) was found to contain mainly compounds such as fatty acid derivatives and terpenoids. The main constituents identified with gas chromatography–mass spectrometry (GC–MS) were (E)-2-hexenal (19%), linalool (9%), 1-hexanol (6.6%), trans-dehydroxylinalool oxide (5.2%), and hexahydrofarnesyl acetone (4.9%). Among the total identified compounds, apocarotenoids accounted for 10.8%, oxygenated monoterpenes for 33.0%, sesquiterpene hydrocarbons for 1.8%, oxygenated sesquiterpenes for 10.3%, oxygenated diterpenes for 3.2%, and non-terpene constituents for 35.8%. Some reports additionally identified cardiac glycosides, phytates, and oxalates. Most of the available information regarding the chemical composition of the leaves, pods, seeds, and roots relied on qualitative chemical reactions for identifying groups of substances, as well as on colorimetric and gravimetric methods of analysis. At this point, there is insufficient quantitative data to precisely determine which anti-nutrients are present in the highest concentrations. Variability in phytochemical profiles appears to be related to ecological factors, genotype, leaf maturity, and extraction methodology.

#### 3.1.2. Seeds

**Figure 3:**
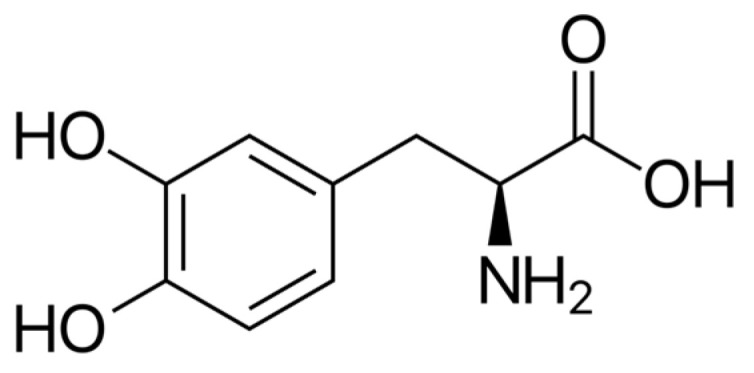
Chemical structure of L-DOPA ((*S*)-2-Amino-3-(3,4-dihydroxyphenyl)propanoic acid).

The findings presented in Table 2 demonstrate that the seeds exhibit the most complex phytochemical composition and consistently contain L-DOPA (Figure 3) as the major bioactive constituent. Based on the available literature, the L-DOPA content in the seeds ranges from 1.79% to 7%. Studies also reported phenolic acids (gallic, caffeic, ferulic, *p*-coumaric, and chlorogenic), flavonoids (quercetin and kaempferol derivatives), tannins, saponins, phytates, raffinose family oligosaccharides, trypsin inhibitors, and cyanogenic compounds. Quantitative variability across studies suggests a strong influence of environmental conditions, processing, and extraction solvents.

#### 3.1.3. Roots

**Figure 4:**
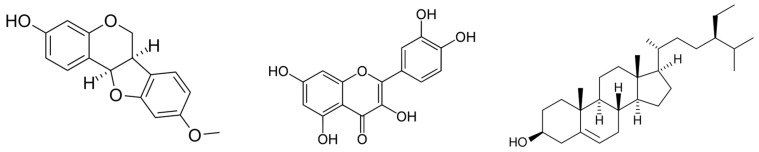
Chemical structures of medicarpin, quercetin, and β-sitosterol detected in the roots.

As shown in Table 3, root extracts contained a narrower but pharmacologically relevant range of compounds, including isoflavones (formononetin), isoflavanones, pterocarpans (maackiain and medicarpin), phenolics, alkaloids, and small amounts of L-DOPA. The chemical structures of representative root-derived compounds, including medicarpin, quercetin, and β-sitosterol, are presented in Figure 4. The limited number of available studies, together with variability in extraction solvents and analytical methodologies, precludes definitive conclusions regarding the chemotaxonomic and therapeutic significance of root-derived metabolites.

#### 3.1.4. Pods

The phytochemical profiles summarized in Table 4 indicate that pod extracts included phenolic compounds, flavonoids, sterols, catechins, L-DOPA, and various minor constituents. Although pods were less chemically diverse than seeds, the presence of antioxidant and anti-inflammatory metabolites indicates potential for further valorization. Differences observed among studies with respect to compound detection and abundance may reflect variability in pod maturity, environmental growth conditions, post-harvest handling, and extraction methodologies.

### 3.2. Biological Activity

#### 3.2.1. In Vitro Studies

In vitro studies (Table 5) demonstrated broad-spectrum antimicrobial, antioxidant, cytoprotective, anti-inflammatory, antifungal, antidiabetic, ACE-inhibitory, and neuroprotective activities. Seed extracts exhibited notable antibacterial effects against *Staphylococcus*, *Shigella*, *Salmonella*, *Vibrio*, and *Escherichia coli.* The antimicrobial potential was assessed using the disk diffusion method. Gentamicin was employed as the positive control to validate the assay [46].

Another study shows that an extract obtained from the seeds exhibits significant antibacterial activity against *Bacillus cereus*, *Staphylococcus aureus*, *Escherichia coli*, *Pseudomonas aeruginosa*, *Klebsiella pneumoniae*, and *Salmonella typhi* [47]. This reveals that *M. pruriens* could be included in future cosmetic or pharmaceutical products for topical application. Leaf extracts displayed antifungal potential, while peptide fractions derived from seed proteins retained activity after simulated digestion [48]. DNA protection, acetylcholinesterase inhibition, and anticancer effects were also documented. Extracts rich in phenolics and L-DOPA consistently showed high antioxidant capacity [49]. An in vitro investigation revealed promising effects in breast cancer, including the induction of DNA damage, stimulation of apoptosis, and other mechanisms. *M. pruriens* also lowered prolactin expression. Hyperprolactinemia associated with cancer leads to resistance to cisplatin (a drug used to treat tumors), and reducing prolactin increases the activity of cisplatin after combination therapy for breast cancer [50]. Another promising application of *M. pruriens* is its use in the treatment of skin diseases. In vitro studies have shown that many skin conditions, such as psoriasis, dermatitis, and eczema, associated with oxidative stress caused by environmental factors like ultraviolet radiation, can be treated. They have shown that the methanol extract from *M. pruriens* leaves has a positive effect on human skin cells (keratinocytes). Specifically, treatment led to a decrease in total protein expression and significantly reduced baseline levels of 4HNE, a marker of oxidative damage [51].

**Table 5:** In vitro studies on *M. pruriens* grouped by primary biological activity.

Study Design	Main Findings	Ref.
Antioxidant and cytoprotective activity		
Protein hydrolysates and peptide fractions were tested under simulated digestion for antioxidant and protective activities.	Peptides remained bioactive after digestion and retained protective effects.	[52]
Phenolic content and antioxidant activity were evaluated using phosphomolybdenum, DPPH, and ABTS assays.	Demonstrated significant antioxidant potential.	[53]
Enzymatically hydrolyzed protein fractions tested on HeLa cells.	Showed notable antioxidant and cytoprotective effects.	[54]
Extracts evaluated for phenolic content, DNA protection, and enzyme inhibition.	Strong antioxidant activity and excellent DNA protection.	[55]
Cotyledon powder tested for DNA protection and copper chelation.	Protected DNA and chelated copper dose-dependently.	[56]
Antimicrobial activity		
Antiviral activity tested on influenza-infected MDCK cells.	Methanol extract showed anti-influenza activity (EC50 32–46 µg/mL).	[57]
Leaf extracts were tested against fungal pathogens via disk diffusion.	Clear antifungal activity (8–20 mm inhibition zones).	[58]
Seed extracts tested against bacteria and *Candida*.	Broad antimicrobial activity except MDR Klebsiella.	[59]
Activity against *Ichthyophthirius multifiliis*.	100% parasite mortality at 150 mg/L.	[60]
Lectins tested against fungal species.	Strong activity against *Candida parapsilosis* only.	[61]
Neuroprotective and enzyme inhibitory activity		
Neuroprotection and acetylcholinesterase inhibition in P19 neuronal cells.	Significant neuroprotection and AChE inhibition.	[49]
Leaf extract evaluated for AChE and lipase inhibition and cytotoxicity.	Strong lipase inhibition; weaker AChE activity.	[62]
Immunomodulatory activity		
Lymphocyte proliferation was assessed with different extracts.	Aqueous and hydroalcoholic extracts stimulated lymphocytes at high concentrations.	[63]
Anticancer and antiproliferative activity		
Methanolic extracts were tested on COLO-205 colorectal cancer cells.	Growth inhibition and apoptosis induction were observed.	[64]
Plant-modified nanoparticles tested on breast cancer cells.	Demonstrated synergistic antitumor effects.	[50]
Cardiovascular-related activity (ACE inhibition, platelets, and cholesterol)		
Peptides from tempeh, a traditional fermented food product, were evaluated for ACE inhibition after digestion.	Highest ACE inhibition after 72 h fermentation.	[65]
Peptide fractions tested for ACE inhibition, antioxidant activity, platelet aggregation, and cholesterol lowering.	Distinct fractions showed cardiovascular benefits.	[66]
Hematological effects		
Extract tested on blood from sickle cell patients.	Improved hemoglobin solubility and reduced osmotic fragility.	[67]
Molecular and mechanistic studies (in silico/pathway interaction)		
Compounds evaluated for interaction with the Wnt/β-catenin pathway using docking and ADME/T.	Gallic acid and other compounds showed strong interactions.	[68]
Enzyme inhibition related to reproductive physiology		
ROCK2 inhibition assay for erectile dysfunction relevance.	The aqueous extract showed inhibitory activity.	[69]
Antivenom-related enzymatic inhibition		
Glutathione tested against snake venom PLA_2_ enzymes.	Strong non-competitive inhibition observed.	[70]

#### 3.2.2. In Vivo Studies

A total of 23 in vivo studies were identified (Table 6). Most notably, extracts demonstrated pronounced antioxidant, anti-inflammatory, analgesic, aphrodisiac, antidiabetic, hepatoprotective, neuroprotective, antihypertensive, immunomodulatory, and antivenom effects across different experimental models.

The enhancement of sexual function is mediated by multiple concurrent mechanisms. One key pathway involves stimulation of endothelial and neuronal nitric oxide synthase (eNOS and nNOS), increasing nitric oxide (NO) production—an effect similar to sildenafil. Elevated NO maintains intracellular cGMP levels, leading to smooth muscle relaxation, vasodilation, and penile erection. Additionally, the extract helps the preservation and restoration of the structure of the corpus cavernosum by improving collagen fibers and sinusoidal spaces, which are often damaged under diabetic conditions [71].

**Figure 5:**
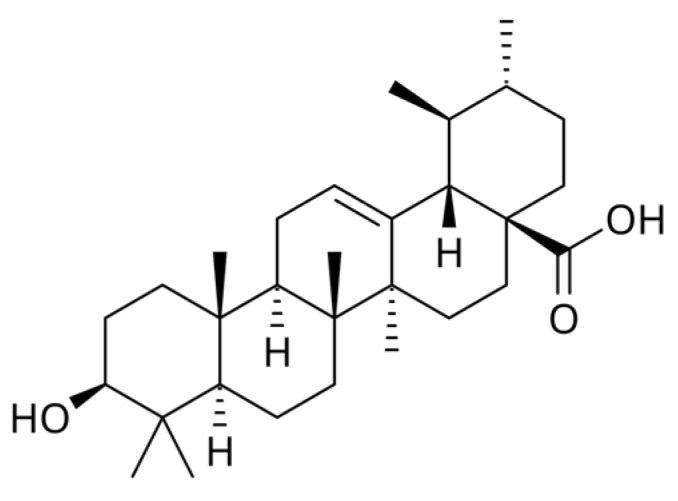
Chemical structure of ursolic acid (3β-Hydroxyurs-12-en-28-oic acid).

**Figure 6:**
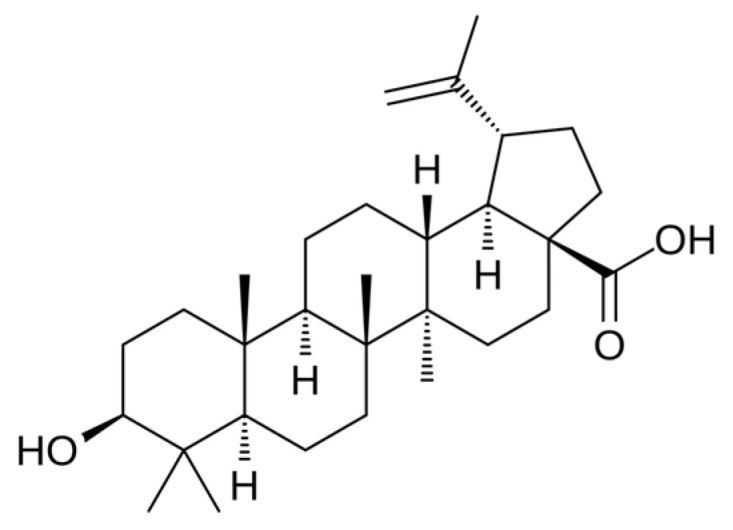
Chemical structure of betulinic acid (3β-Hydroxylup-20(29)-en-28-oic acid).

Neuroprotective effects included preservation of dopaminergic neurons, reduced oxidative stress, and improved behavioral outcomes in models of Parkinson’s and Huntington’s disease. This activity is also attributed to ursolic acid (Figure 5) and betulinic acid (Figure 6), pentacyclic triterpene saponins with antioxidant and anti-inflammatory properties.

Their anti-inflammatory action involves inhibition of NF-κB nuclear translocation, reducing the expression of pro-inflammatory cytokines such as TNF-α and enzymes like inducible nitric oxide synthase. Additionally, they prevent neuronal apoptosis by increasing anti-apoptotic proteins (Bcl-2, pAkt1), decreasing pro-apoptotic Bak, and improving mitochondrial function [72].

**Table 6:** Summary of in vivo studies on *M. pruriens* grouped by primary biological activity.

Study Design	Main Findings	Ref.
Neuroprotective and CNS effects		
Twenty-four Swiss albino mice were assigned to three groups: Control, Paraquat (PQ), and PQ + *M. pruriens*. PQ was administered intraperitoneally twice per week, while the ethanolic seed extract was given orally for nine weeks. Nitrite levels and lipid peroxidation were assessed.	*M. pruriens* provided protective effects on dopaminergic neurons against NO-induced damage in the substantia nigra.	[73]
Forty-eight Wistar rats were randomly assigned to four groups to evaluate neuroprotective effects in cerebral ischemia.	Extract and β-sitosterol reduced N-methyl-D-aspartate receptor and tau expression, decreasing ischemic damage.	[74]
Parkinson’s disease induced by rotenone in mice followed by treatment with extract or L-DOPA.	Extract was as effective as synthetic L-DOPA in improving motor and neuroinflammatory outcomes.	[75]
6-Hydroxydopamine lesion model in Sprague-Dawley rats.	Powder restored neurotransmitter levels and showed strong neuroprotection.	[76]
3-Nitropropionic acid Huntington-like model in rats.	Extract reduced oxidative stress and neuroinflammation and preserved neuronal integrity.	[77]
Tetrahydroisoquinoline was tested in *C. elegans* and Parkinson’s disease rat models	Compound enhanced L-DOPA effects and improved motor function.	[78]
Reproductive and sexual function		
Male Wistar rats were evaluated for mounting, intromission, and ejaculation parameters.	Extract significantly enhanced sexual behavior.	[79]
Long-term administration assessing testosterone and penile reflexes.	Improved testosterone levels and nerve integrity.	[71]
Diabetic rats were treated with the extract or sildenafil.	Restored erectile tissue integrity via antioxidant effects.	[80]
Rats treated with extract (250–500 mg/kg) to assess sexual behavior and glucose.	Reduced glucose and improved sexual performance.	[81]
Metabolic and antidiabetic effects		
Corn starch load model assessing glucose response.	Significant antihyperglycemic activity via α-glucosidase inhibition.	[82]
Wistar rats with induced diabetes were used to evaluate the effects of fermented velvet bean seeds.	Improved glycemic control, lipid profile, and antioxidant status.	[83]
Cardiovascular and antihypertensive activity		
Mean arterial pressure measured in anesthetized rats.	Extract reduced mean arterial pressure (MAP) and enhanced bradykinin hypotension.	[84]
Nω-nitro-L-arginine methyl ester (L-NAME) hypertension model in rats.	Protein hydrolysates reduced systolic and diastolic pressure.	[85]
Hepatoprotective and organ-protective effects		
Ethanol and antibiotic-induced liver injury model.	Extract reduced liver enzymes and bilirubin.	[86]
Toxicity and safety		
Acute and sub-acute toxicity in mice and rats (up to 8 g/kg).	No toxicity observed, organs unaffected.	[19]
30-day oral administration assessing liver markers.	No toxic effects; enzymes within normal range.	[87]
Anti-inflammatory and analgesic effects		
Essential oil tested in hot plate, formalin, and carrageenan models.	Strong analgesic and anti-inflammatory activity.	[9]
Immunomodulatory activity		
SRBC antibody response in mice.	Increased antibody titers and leukocyte mobilization.	[88]
Wound healing		
Hydrogel evaluated in excision, incision, and dead space wounds.	Significant wound healing effects.	[89]
Antiparasitic, antivenom, and other effects		
Lamb infection model with dietary supplementation.	No significant antiparasitic effect.	[90]
Venom protection model in mice.	Protective effect only with pre-treatment.	[91]
DMBA tumor model in female rats.	Reduced DNA damage and tumor-related changes.	[92]

In vivo investigation with the plant’s seeds showed positive results against neurotoxins, cardiotoxins, cytotoxins, phospholipase A_2_ (PLA_2_), and proteases contained in snake venom [91]. In Nigeria, the plant is widely used as a means of prevention and protection, with its seeds being prescribed prophylactically for ingestion. It is claimed that this protects humans from the effects of snake bites for a whole year [91]. One study investigated the effect of photo biomodulation on histamine and itching caused by *M. pruriens* [93]. The results of this study were not analyzed, as they were not directly related to the therapeutic potential of the plant. The plant showed promising potential to be studied in more detail for possible inclusion in the treatment of Parkinson’s disease. Parkinson’s disease is a condition characterized by decreased dopamine levels. The change in dopamine levels may be due to the progressive loss of dopaminergic neurons in the substantia nigra pars compacta [94]. Due to their L-DOPA content, *M. pruriens* seed extract intranasal gel has been used in animals for the management of Parkinson’s disease. According to this study, the dopamine levels were significantly increased [95].

#### 3.2.3. Clinical Studies

Human studies remain limited (Table 7) but provide encouraging preliminary evidence. Preparations derived from *M. pruriens* seeds improved motor function and reduced dyskinesia in Parkinson’s disease patients, often with faster onset of action compared with standard levodopa formulations. Gastrointestinal discomfort was the most frequently reported adverse effect [11]. In studies involving infertile men, seed supplementation improved semen volume, sperm concentration, motility, hormonal balance, and oxidative stress markers [96].

### 3.3. Toxicity

Despite its high nutritional value, certain parts of *M. pruriens* may pose significant health risks. In addition to its content of vitamins, amino acids, and various micro- and macronutrients, the plant also contains several toxic principles. Some anti-nutrients, such as tannins, oxalates, cyanides, and saponins found in the leaves, as well as L-DOPA present in the seeds and roots, have the potential to exert adverse effects on health.

Evidence indicates that the levels of these compounds in the leaves are generally low and unlikely to cause significant harm. The seeds, however, present a greater toxicological concern due to their high L-DOPA content. When combined with other anti-nutrients, L-DOPA has been shown in in vivo studies in rats to induce pathological changes in multiple organs, including the lungs, heart, kidneys, liver, and brain [101].

The most common side effects of *M. pruriens* are diarrhea, nausea, vomiting, bloating, headaches, dizziness, insomnia, anxiety, and low blood pressure. Aside from the seeds, adverse effects may also arise from the pods, which are densely covered with hairs. The limited studies available on the pods indicate that they contain compounds capable of inducing redness and severe, painful itching. Evidence suggests that this reaction is triggered by a novel cysteine protease, identified as mucunain. Mucunain activates protease-activated receptors (PARs), specifically PAR2 and PAR4. The activation of these receptors leads to mobilization of intracellular calcium, resulting in severe itching [102].

However, another in vivo investigation on the guinea pig’s ileum discovered that it is not mucunain that causes this itching effect but histamine and its analogs [103]. The underlying mechanism may arise either from histamine binding directly to its H1 receptors or from contact-induced activation of mast cells by active principles, which rapidly trigger mast cells to release histamine and subsequently cause itching and rash [4].

In some in vivo studies in rats, the highest administered dose has reached 2000 mg/kg body weight without severe toxicity being reported. In other studies, the highest dose administered to humans was 30 g seed powder, which contained up to 1000 mg neat L-DOPA. At this dose, adverse effects were generally mild and short-lived [99,104]. In the absence of peripheral decarboxylase inhibition, systemic availability of levodopa is expected to be low, and potential matrix effects of the plant preparation require further controlled investigation.

## 4. Discussion

This review summarizes current knowledge on the phytochemical composition and biological activity of *M. pruriens*, highlighting its therapeutic significance and gaps in scientific research. The plant exhibits considerable variability in the chemical composition of its various morphological organs, with the seeds being the most widely characterized part due to their high L-DOPA content [34]. In contrast to L-DOPA, dopamine itself cannot cross the blood–brain barrier, as the enzyme dopa decarboxylase within peripheral tissues rapidly metabolizes it before it reaches the brain [13]. The diversity of metabolites documented in the seeds determines their importance as the most pharmacologically significant part of the species studied and justifies further studies of bioavailability, pharmacodynamics, and safety profiles in both preclinical and clinical contexts.

The characteristic presence of fatty acid derivatives, terpenoids, and phenolic compounds in the plant’s essential oils supports the observed antioxidant, antimicrobial, and anti-inflammatory properties reported in numerous in vitro and in vivo models. This composition is consistent with the traditional use of the plant in folk medicine, especially in regions such as Nigeria, Ghana, and India, where *M. pruriens* is used as a tonic for the nervous system [9].

As indicated by the studies summarized in Table 1, maceration of leaf material in various solvents is the most employed method for extracting phytochemicals. In those studies, in which the percentage content is indicated, methanol appears to provide the richest chemical profile. Similarly, phytochemical extraction from seeds, roots, and pods is predominantly performed via maceration using different organic solvents [17]. As shown in Table 2, Table 3 and Table 4, the extraction yield varies slightly depending on the solvent employed. Polar organic solvents tend to enrich phenolics and certain anti-nutritional factors, while other protocols may yield preparations with different L-DOPA or protein content. However, the considerable variability in phytochemical profiles across studies underscores the need for standardized cultivation, harvesting, and extraction protocols. This will enable robust comparisons and facilitate the identification of reliable chemical markers for quality assessment, as well as justify further research through quantitative phytochemical analyses and targeted studies of biological activity.

Based on the currently available evidence, future pharmacological and clinical studies on *M. pruriens* would benefit from standardized preparations. Seed extracts should be prioritized, as the seeds consistently contain high and quantifiable levels of L-DOPA, which is directly linked to the plant’s neuroprotective and clinical effects [49]. Hydroalcoholic or aqueous seed extracts appear most appropriate, given their frequent use in experimental and human studies. In addition to L-DOPA as the primary chemical marker, selected phenolic compounds (gallic acid, ferulic acid, and quercetin) or total phenolic content could serve as complementary markers to reflect antioxidant and anti-inflammatory activity [21]. Such a combined standardization approach may improve reproducibility, batch-to-batch consistency, and translational relevance in future controlled pharmacological and clinical investigations.

The wide range of biological activities documented in vitro and in vivo confirms many of the plant’s traditional uses. The neuroprotective effects, particularly important in Parkinson’s disease, appear to result from the synergistic action of L-DOPA, phenolic antioxidants, such as flavonoids (quercetin and kaempferol derivatives, catechins, and rutin), phenolic acids (gallic, caffeic, ferulic, *p*-coumaric, and chlorogenic), and other neuroactive metabolites [76]. Evidence also supports the plant’s role in male reproductive health, with improvements in hormonal profile and sperm parameters attributed to catecholamine restoration and antioxidant effects. The secondary metabolites, such as polyphenols and glutathione, are known for their high antioxidant activity and scavenge free radicals, thereby inhibiting oxidative processes [105].

From a pharmacological point of view, the high content of L-DOPA is a key factor distinguishing *M. pruriens* from other medicinal plants. The presence of this direct precursor of dopamine provides a biological basis for the use of the plant in diseases characterized by dopaminergic dysfunction, such as Parkinson’s disease [13,98]. Data from experimental models show that, in addition to restoring dopaminergic tone, the plant exerts neuroprotection through several mechanisms: reduction in oxidative stress, improvement in mitochondrial function, and suppression of neuroinflammatory processes. These effects support the concept of multimodal action of the plant on the central nervous system [49,76].

In vitro results demonstrate a wide range of biological activities, including antioxidant, antibacterial, antifungal, antiviral, antiparasitic, and antitumor effects. It is important to note that a significant portion of the reported effects, including cytoprotective and DNA-protective activities, are associated with high concentrations of phenolic and dopaminergic metabolites [55]. In addition, a considerable number of studies support the plant’s potential as a stimulant of sexual function and fertility, which is consistent with its traditional ethnomedical applications.

Studies involving Wistar rats have indicated that the ethanolic extract of *M. pruriens* exhibits dose- and time-dependent blood glucose-lowering effects. At higher doses (above approximately 50 mg/kg crude or partially characterized extracts), its activity approaches that of standard antidiabetic agents. Additionally, the extract appears to counteract diabetes-related weight loss, suggesting possible benefits in maintaining body mass during diabetic conditions [106].

This antidiabetic effect of *M. pruriens* results from multiple simultaneous mechanisms. These include inhibition of α-amylase and α-glucosidase, which reduces intestinal glucose absorption, and the indirect stimulation of insulin secretion by minerals (e.g., zinc and magnesium) and bioactive compounds such as L-DOPA, flavonoids, phenols, and tannins [107]. However, most preclinical antidiabetic studies use non-standardized extracts, limiting interpretation of dose-response relationships and preventing accurate estimation of exposure to L-DOPA or specific phenolics. Future studies should report chemical standardization to enable meaningful comparisons. One of the most pronounced activities associated with *M. pruriens* is its libido-enhancing potential. It was demonstrated both in animal (Table 6) and human studies (Table 7). An in vivo investigation found that men with reduced sperm count or motility exhibited notably lower seminal plasma concentrations of dopamine, adrenaline, and noradrenaline, along with decreased serum testosterone and luteinizing hormone levels [100]. Conversely, prolactin and follicle-stimulating hormone were elevated compared with values observed in healthy controls. Administration of *M. pruriens* has been shown to restore catecholamine levels and improve hormonal balance by increasing testosterone and luteinizing hormone, while lowering prolactin and follicle-stimulating hormone [100]. This effect is attributed to the high L-DOPA content. L-DOPA exerts a direct influence on the hypothalamic–pituitary–gonadal (HPG) axis, leading to increased secretion of gonadotropin-releasing hormone (GnRH), luteinizing hormone (LH), and enhanced testosterone production [100]. This hormonal normalization is associated with enhanced sexual drive. Reported outcomes include improved erection, mounting frequency, intromission frequency, mounting latency, intromission latency, ejaculation latency, post-ejaculatory interval, and ejaculation frequency, as well as elevations in dopamine levels [100,108]. However, antioxidant constituents may also contribute by reducing oxidative stress and improving sperm function, and current clinical evidence does not fully disentangle L-DOPA-dependent effects from those of other metabolites.

Moreover, *M. pruriens* appears to have potential as a fertility-enhancing agent. Infertility is an emerging condition that is increasingly affecting couples worldwide and has therefore attracted growing scientific attention. Research investigating the effects of *M. pruriens* seeds in relation to this condition has advanced substantially, with recent studies progressing toward in vivo evaluation [100].

In vivo studies confirm many of these observations but also highlight certain limitations. The reported antioxidant, antidiabetic, neuroprotective, and reproductive effects show dose- and time-dependent effects, suggesting a need for standardized extracts and optimized dosing regimens. However, significant differences in the extraction methods used, plant parts, dosages, and duration of treatment make it difficult to directly compare individual studies. The small sample sizes in animal models further limit the ability to draw general conclusions about safety and long-term effects.

Clinical data, although limited, suggest that preparations of *M. pruriens* with a high content of natural L-DOPA may provide motor benefits comparable to or even more favorable than standard levodopa preparations, including a faster onset of action and a lower incidence of dyskinesia in some patients [11]. Additional clinical observations in men with infertility support the hypothesis that the plant improves hormonal balance, reduces oxidative stress, and optimizes semen quality [96]. However, the lack of well-controlled, randomized studies with sufficiently large cohorts limits the ability to definitively confirm these effects.

Despite promising results, the translation of preclinical evidence into clinical practice remains limited. Human studies are few, often with small samples, short duration, and heterogeneous methodologies. Furthermore, concerns about tolerability, particularly gastrointestinal side effects, highlight the need for optimized formulations to improve patient compliance [104].

Toxicological data suggest that the plant is generally safe, and high doses have been reported as tolerated when consumed in a processed form. Defining a clear therapeutic margin is challenging due to variability in L-DOPA content and the presence of anti-nutritional factors such as tannins and saponins. The high L-DOPA content in the seeds and irritating compounds in the pod hairs warrant caution. Potential risks may relate to excessive dopaminergic stimulation as well as gastrointestinal or metabolic effects associated with these constituents, highlighting the need for careful dose selection and standardized preparations, particularly for chronic use. There is a lack of data on long-term safety and analyses of potential interactions with conventional medications.

## 5. Conclusions

*M. pruriens* is an important medicinal species with a long history of traditional use and a growing body of scientific evidence supporting its therapeutic potential. It is characterized by a complex and organ-specific phytochemical profile. Among its constituents, L-DOPA in the seeds represents the most clinically significant compound and provides a clear mechanistic basis for the plant’s established and emerging role in the management of Parkinson’s disease. In addition to its dopaminergic activity, preclinical evidence supports antioxidant, anti-inflammatory, metabolic, hepatoprotective, antimicrobial, and reproductive effects, likely mediated by phenolic compounds, flavonoids, alkaloids, and other secondary metabolites.

Despite extensive in vitro and in vivo investigations, translation into clinical practice remains limited. Available human studies, although encouraging, are few and frequently involve small sample sizes and heterogeneous methodologies. Substantial variability in plant part selection, extraction procedures, phytochemical characterization, and dosing regimens further complicates direct comparison between studies and limits definitive therapeutic positioning.

Toxicological data suggest generally favorable tolerability, particularly when seeds are properly processed. However, the high and variable L-DOPA content and the presence of anti-nutritional or irritant compounds necessitate careful standardization and dose consideration, especially for long-term use.

Future research should prioritize standardized cultivation and extraction protocols, quantitative phytochemical profiling with defined chemical markers, and well-designed, adequately powered clinical trials. Such efforts are essential to establish reproducible efficacy, clarify safety margins, and support the evidence-based integration of *M. pruriens* into contemporary phytopharmacotherapy.

## Figures and Tables

**Table 1:** Chemical composition of *M. pruriens* leaf extracts.

Country of Origin	Extraction Method	Main Compounds Identified	Ref.
Nigeria	Hydrodistillation	(*E*)-2-hexenal, linalool, 1-hexanol, trans-dehydroxylinalool oxide	[9]
Brazil	Methanol maceration	Alkaloids, anthraquinones, phenolics, coumarins, flavonoids (quercetin, rutin), saponins, tannins	[17]
Indonesia	Microwave-assisted extraction	Inositol, butanedioic acid, *D*-gluconic acid, anthraquinone, isoquinoline	[18]
Nigeria	Water maceration	Carbohydrates, anthraquinones, saponins, cardiac glycosides, flavonoids	[19]
Nigeria	Cold/hot aqueous maceration	Phytate, oxalate, tannins, saponins, hydrocyanic acid	[20]
Bangladesh	Methanol maceration	Phenolics, flavonoids, ferulic acid, benzofuran derivative, stizolamine	[21]

**Table 2:** Chemical composition of *M. pruriens* seed extracts.

Country of Origin	Extraction Method	Main Compounds Identified	Ref.
Saudi Arabia/India/Republic of Korea	Chloroform–methanol extraction	Hydroxylamine (88.00%); trichloromethane (1.1%); 2-ethyl-1-hexanol (0.15%); asarone (0.38%); phthalic acid, butyl hept-3-yl ester (11.00%)	[22]
Cameroon	Water maceration	L-DOPA, polyphenols, flavonoids, tannins, phytates	[23]
Nigeria	Water maceration	Flavonoids, alkaloids, saponins, tannins, cyanide, phenol, phytates	[24]
India	NaHCO_3_ maceration	Tannins, L-DOPA, phytic acid, raffinose, stachyose, verbascose, enzyme inhibitors	[25]
India	Milk maceration	Phenolic compounds, L-DOPA, tannins, flavonoids, phytates, oxalates	[26]
Germany	Water/alkaline maceration	L-DOPA, phenolics, tannins, phytates, saponins, trypsin inhibitors	[27]
India	Soxhlet (petroleum ether)	L-DOPA, phenolics, tannins, trypsin inhibitors	[28]
Nigeria	Water maceration	Tannins, phytin, lectin, cyanide, enzyme inhibitors	[29]
Brazil	Hydroalcoholic extract	Flavonoids, saponins, tannins, sterols	[30]
Nigeria	Multiple methods	L-DOPA, oxalate, phytate, cyanide, phenols, tannins, saponins	[31]
Nigeria	Multiple methods	Tannins, L-DOPA, phytic acid, saponins, polyphenols, oligosaccharides	[32]
Nigeria	Hydroalcoholic extract	Total phenolics, flavonoids, proanthocyanidins	[33]
Thailand	Soxhlet (ethanol)	L-DOPA, gallic acid, quercetin, propanetriol, monoacetate, butyl 2-methyl butanoate, palmitic acid, linoleic acid, stearic acid, 4-hydroxy-2,2,6-trimethylcyclohex-2-enone, 2-ethylacridine, bis(2-ethylhexyl) phthalate	[34]
India	Acetonitrile–methanol ultrasonic extraction	Total phenolic content, total flavonoid content, tannin, phytic acid	[35]
India	Soxhlet (methanol)	L-DOPA, phenolics, flavonoids, tannins, fatty acids	[36]

**Table 3:** Chemical composition of *M. pruriens* root extracts.

Country of Origin	Extraction Method	Main Compounds Identified	Ref.
Thailand	Maceration of the roots in methanol and dichloromethane	Parvisoflavanone, lespedeol, uncinanone, (*6aR*,*11aR*)-medicarpin, maackiain, formononetin	[37]
India/Oman	Soxhlet procedure with methanol	Phenols (gallic acid), flavonoid (quercetin), glycosides, steroids, alkaloids	[38]
India	Maceration of the roots in a mixture of chloroform and ammonia	*N*,*N*-dimethyltryptamine-Nb-oxide, 5-methoxy-N, *N*-dimethyltryptamine, bufotenine, serotonin, choline, unidentified β-carboline, unidentified indole-3-alkylamines	[39]
India	Extraction with methanol under reflux on a water bath	β-sitosterol (0.076%)	[40]
India	Maceration of the roots in methanol	L-DOPA, carbohydrates, proline, phenolics, flavonoids	[41]
The Netherlands	Acidification method with 5% formic acid	L-DOPA	[42]

**Table 4:** Chemical composition of *M. pruriens* pod extracts.

**Country of Origin**	**Extraction Method**	**Main Compounds Identified**	**Ref.**
Argentina/ Uruguay	Ultrasound-assisted extraction/decoction extraction	L-DOPA (5.80%—pods, and 9.50%—seeds), ferulic acid, ursolic acid, catechin, quercetin, gallocatechin, chlorogenic acid, gallocatechin gallate, stigmasterol, daucosterol, glutathione, 6-methoxyharman, daidzein, prunetin, procyanidin dimer, serotonin, kaempferol, b-sitosterol	[43]
Argentina	Maceration of the pods in a mixture of water, 50% methanol, 100% methanol, 50% ethanol, and 100% ethanol/Decoction in boiling water	Total phenolic (gallic acid), total flavonoid (quercetin), total ortho-diphenol content (caffeic acid)	[44]
USA	Soxhlet procedure with ether	Total phenolics, L-DOPA, tannins	[45]

**Table 7:** Human studies on *M. pruriens* grouped by primary biological activity.

Study Design	Main Findings	Ref.
Antiparkinsonian activity		
This study evaluated the efficacy and safety of a single dose of *M. pruriens* powder prepared from roasted seeds without pharmacological processing. Eighteen patients with advanced Parkinson’s disease received six randomized treatments, including varying doses, levodopa formulations, and placebo.	Compared with levodopa plus benserazide, low-dose *M. pruriens* produced a similar motor response with fewer side effects, while a high dose yielded greater motor improvement, longer “on” duration, and fewer dyskinesias. Adverse events were minimal, and tolerability was better than standard levodopa.	[10]
Eight patients with Parkinson’s disease participated in a randomized, double-blind, controlled crossover study. Each received single doses of levodopa/carbidopa (200/50 mg) and 15 g or 30 g *M. pruriens* seed powder at one-week intervals.	The 30 g preparation produced a significantly faster onset of action compared with levodopa/carbidopa (34.6 vs. 68.5 min; *p* = 0.021), corresponding to quicker peak plasma L-DOPA levels.	[11]
Fourteen Parkinson’s disease patients participated in a 16-week randomized crossover trial comparing *M. pruriens* powder with levodopa/carbidopa. Outcomes included quality of life, motor and non-motor symptoms, and safety parameters.	Half discontinued *M. pruriens* due to gastrointestinal discomfort or worsening symptoms. Among those who tolerated treatment, efficacy was comparable to levodopa/carbidopa across outcome measures.	[97]
A 12-week open-label trial evaluated HP-200, a *M. pruriens* preparation, in 60 Parkinson’s patients (levodopa-naive and previously treated).	Significant improvements in Hoehn and Yahr staging were observed. Side effects were mild (mainly gastrointestinal), with no laboratory abnormalities.	[98]
Twelve participants received 30 g *M. pruriens* powder and levodopa (100/25 mg × 2) in separate sessions with pharmacokinetic assessment.	*M. pruriens* showed higher systemic exposure and prolonged ON time without dyskinesia (232.2 vs. 161.8 min), indicating greater bioavailability.	[99]
Male fertility and reproductive effects		
A study of 150 men (fertile controls and infertile subgroups) assessed the effects of 5 g/day *M. pruriens* seed powder for three months. Semen and blood parameters were measured before and after treatment.	Treatment increased testosterone, LH, dopamine, adrenaline, and noradrenaline, while reducing FSH and prolactin. Significant improvements in sperm count and motility were observed.	[100]
A study including 120 participants (fertile controls and infertile subgroups) evaluated 5 g/day seed powder for three months with biochemical and semen analysis.	Treatment improved sperm count and motility, reduced lipid peroxidation, restored antioxidant levels, and corrected seminal plasma parameters, supporting restorative effects in infertility.	[96]

## Data Availability

Data available in the article.
